# The Suitability of Orthogonal Hosts to Study Plant Cell Wall Biosynthesis

**DOI:** 10.3390/plants8110516

**Published:** 2019-11-17

**Authors:** Markus Pauly, Niklas Gawenda, Christine Wagner, Patrick Fischbach, Vicente Ramírez, Ilka M. Axmann, Cătălin Voiniciuc

**Affiliations:** 1 Institute for Plant Cell Biology and Biotechnology, Heinrich Heine University Düsseldorf, 40225 Düsseldorf, Germany; m.pauly@hhu.de (M.P.); Niklas.Gawenda@uni-duesseldorf.de (N.G.); ramirezg@hhu.de (V.R.); 2 Independent Junior Research Group–Designer Glycans, Leibniz Institute of Plant Biochemistry, 06120 Halle (Saale), Germany; Christine.Wagner@ipb-halle.de; 3 Institute of Synthetic Biology, Heinrich Heine University Düsseldorf, 40225 Düsseldorf, Germany; patrick.fischbach@hhu.de; 4 Institute for Synthetic Microbiology, Heinrich Heine University Düsseldorf, 40225 Düsseldorf, Germany; ilka.axmann@hhu.de

**Keywords:** cell walls, polysaccharides, synthetic biology, glycosyltransferases, heterologous expression

## Abstract

Plant cells are surrounded by an extracellular matrix that consists mainly of polysaccharides. Many molecular components involved in plant cell wall polymer synthesis have been identified, but it remains largely unknown how these molecular players function together to define the length and decoration pattern of a polysaccharide. Synthetic biology can be applied to answer questions beyond individual glycosyltransferases by reconstructing entire biosynthetic machineries required to produce a complete wall polysaccharide. Recently, this approach was successful in establishing the production of heteromannan from several plant species in an orthogonal host—a yeast—illuminating the role of an auxiliary protein in the biosynthetic process. In this review we evaluate to what extent a selection of organisms from three kingdoms of life (Bacteria, Fungi and Animalia) might be suitable for the synthesis of plant cell wall polysaccharides. By identifying their key attributes for glycoengineering as well as analyzing the glycosidic linkages of their native polymers, we present a valuable comparison of their key advantages and limitations for the production of different classes of plant polysaccharides.

## 1. Introduction

Plant cells are encapsulated by a sophisticated composite material, the cell wall. The wall consists of various polymer networks encompassing mainly polysaccharides, but also glycoproteins and lignin (a phenolic polymer) [1]. Enormous progress has been made in elucidating the components of the biosynthetic machinery of these cell wall polysaccharides. For example, most of the carbohydrate-active enzymes that play a role in the synthesis of all known plant polysaccharide classes, including cellulose, the hemicelluloses xyloglucan, (hetero)mannan, (hetero)xylan, mixed-linkage glucan, the pectic polysaccharides homogalacturonan, rhamnogalacturonan I and II, and the arabinogalactan proteins and extensin glycoproteins, have now been identified [2,3,4]. While cellulose and callose are synthesized directly at the plasma membrane [5], matrix polysaccharides such as pectins and hemicelluloses are synthesized in the Golgi apparatus and secreted to the extracellular space via vesicles [6,7]. The glycosyltransferases have been identified based on biochemical enrichment strategies, heterologous protein expression followed by in vitro activity assays [2], and/or the isolation of plant mutants [8,9,10,11]—via knock-out, knock-down, and/or overexpression of the corresponding genes—followed by wall polymer analysis of mutant walls. However, even once the substrate specificity of a glycosyltransferase (GT), its enzyme kinetics, and its cellular location are characterized (still a rare occurrence), many questions remain unanswered. For example:

1. What determines the length of a polysaccharide?
2. What determines the substitution patterns of a polysaccharide?
3. What are the roles of auxiliary proteins and/or cofactors in polysaccharide synthesis?
4. How is the product influenced by the supply of activated precursors such as nucleotide sugars?

To address these open issues, it is desirable to go beyond individual GTs and reconstruct an entire biosynthetic machinery required to produce a complete wall polysaccharide. If this reconstruction leads to the synthesis of the exact polysaccharide structure found in a native wall, then one can assume that all factors involved in the process have been identified. However, if for instance the substitution pattern of a polysaccharide differs from that found in the plant wall, then hitherto unidentified or uncharacterized factors are missing and remain to be discovered and characterized.

**Figure 1:**
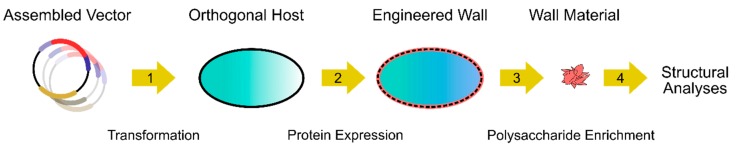
Strategy to study plant cell wall biosynthesis using synthetic biology. Plant genes of interest are assembled in one or more vectors and transformed (Step 1) into orthogonal hosts. Following recombinant protein expression (Step 2), the extracellular matrix of the engineered cells is extracted to assess if the expected polymer has been synthesized (Step 3). Lessons from structural analyses of enriched polysaccharides (Step 4) inform the next round of engineering.

One recent approach that allows for the reconstruction of a whole functional biosynthetic machinery is synthetic biology (Figure 1), whereby multiple genes are expressed in an orthogonal organism that does not contain the polysaccharide structure of interest [12]. The study of GTs in orthogonal unicellular systems provides higher-throughput, faster genetic engineering, and reduced glycan complexity compared to plants. While in vitro assays of purified proteins provide the cleanest background and have been instrumental to elucidating GT activities [2], the synthetic biology strategy outlined in Figure 1 bypasses time-consuming protein extraction and solubilization steps, and offers superior polysaccharide yields. Furthermore, orthogonal hosts could be exploited to produce “fit for purpose” polysaccharides (e.g., for functional foods and fibers) and to monitor their dynamics, two objectives that remain technically challenging in plants [13,14,15]. Plant polysaccharide-based nanocomposites already have a variety of industrial applications [16], including drug delivery, so gaining the ability to tailor glycan structures and attributes is of high biotechnological value.

The synthetic biology approach (Figure 1) has been successful in the reconstruction of plant mannan as well as glucomannan polymers in yeast [17], also illuminating the role of an auxiliary protein in the biosynthetic process. Since land plants and algae share some common cell wall components [18], it is necessary to look beyond the plant kingdom for a cellular host that provides a clean background for polysaccharide production. In this review, we evaluate whether a selection of organisms from three kingdoms of life (Bacteria, Fungi and Animalia) might be suitable for the synthesis of plant cell wall polysaccharides and present their advantages and limitations.

## 2. Criteria for the Choice of Orthologous Hosts

When considering a host for polysaccharide production, one needs to take into account a number of issues. To reduce complexity and increase reproducibility, the most suitable organisms for synthetic biology are unicellular or immortalised cell lines from multicellular organisms (e.g., human embryonic kidney 293 cells, HEK293). Such cells should be amenable to metabolic engineering, convenient, and affordable to cultivate to accumulate biomass for comprehensive cell wall analyses. 

**Figure 2:**
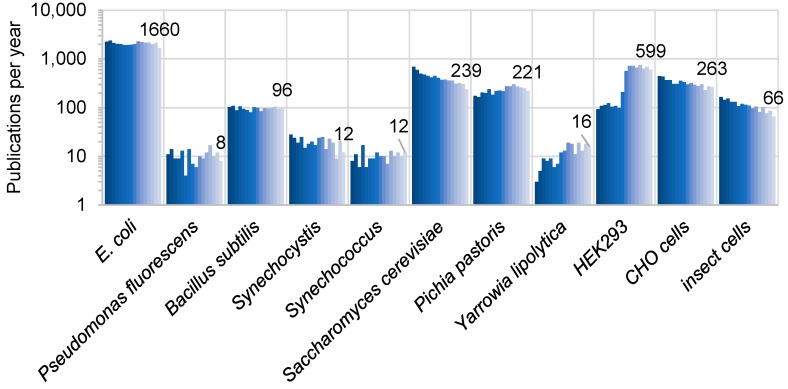
Publications on recombinant proteins in different expression hosts in the last 15 years. The graph shows, as a proxy for the popularity of a particular expression system, the number of publications indexed in the PubMed.gov database from 2003 (dark blue) to 2018 (light blue) that matched the (“X axis label” recombinant protein) search, performed on November 6, 2019. The vertical axis shows a log scale (base 10), and the data labels on the chart represent the 2018 publication count for each host. Only 73 total matches (over 15 years) were found for *Ustilago maydis*, and are thus not shown in the graph.

For biotechnological applications, a variety of bacteria, fungi and animal cell lines have been successfully used to express recombinant proteins. In this review, we focused on 11 potential hosts (Table 1) and assessed the genetic tools available to metabolically engineer these organisms for polysaccharide production. This list is not exhaustive but includes most of the commonly used expression systems with a potential for a synthetic biology approach (Figure 2). For instance, we excluded *Agrobacterium tumefaciens*, which revolutionized plant biotechnology [19], because it has been primarily used as a DNA transfer vehicle and lacks the tools necessary for metabolic engineering. Although proven to produce active cell wall-related GTs (CWGTs) [20], we omitted insect cells from in-depth analysis and focused instead on two mammalian cells lines which are more commonly used for recombinant protein expression (Figure 2). 

Most CWGTs of plant origin are transmembrane proteins that have been historically challenging to express in an active form using orthogonal hosts [2]. Therefore, it is desirable that a candidate organism has already been shown to functionally produce plant GTs. Such data would set a precedent to ensure that the native post-translational modifications (PTMs), or lack thereof, do not interfere with enzymatic activity. Therefore, we summarize in Table 1 which organisms were already successfully used to express active plant CWGTs, and more broadly, other GT classes involved in secondary metabolism. 

Even within a species, genetic diversity and the potential to modulate biochemical pathways is enhanced by availability of different recombinant protein expression strains as well as mutant libraries. As the best eukaryotic example, the *Saccharomyces cerevisiae* Yeast Knockout (YKO) Collection includes >21,000 mutant strains that carry precise start-to-stop deletions of 6000 *Saccharomyces* genes [21]. Such genetic resources enable the use of a single organism to address a greater variety of biological questions, and could be exploited to rapidly identify strains that are advantageous for the production of distinct polysaccharides.

Organisms that natively secrete enzymes capable of degrading plant polysaccharides (e.g., *Aspergillus niger*) are biotechnologically relevant for biomass conversion, so they are of limited value for the production of cell wall polymers [22]. Nevertheless, even plant pathogens can be genetically engineered for such applications. For example, the smut fungus *Ustilago maydis* that infects maize has been genetically engineered to grow in a yeast-like filamentous form unable to infect plant cells [23], which we assessed in more detail for this review. 

Another essential requirement for synthetic biology approaches is the availability of vectors, ideally compatible with cloning techniques based on interchangeable modules such as promoters, transcriptional terminators, and selection markers. Golden Gate cloning is a technology that exploits the ability of Type IIS enzymes (e.g., BsaI and BpiI) to cut outside their recognition site and permits multiple DNA fragments with complementary overhangs (defined by 4 bp fusion sites) to be efficiently assembled in a one-pot reaction [24]. Provided that the starting parts are verified to be correct, vectors assembled using this simple cut-and-paste method do not require further sequencing, thus accelerating the building process. Alternatively, sequence-independent cloning methods such as Gibson Assembly [25] or enzyme-free AQUA cloning [26] can be used to join different DNA fragments seamlessly. Such techniques and the availability of standardized parts from global stock centers (e.g., AddGene, a non-profit repository for plasmids) enable researchers around the world to build multimeric genetic circuits whose individual components can be easily interchanged and tested for optimal results. 

Strong, inducible promoters are ideal to control transgene expression, although promoters of varying strengths can help to fine-tune gene expression. A potential drawback of constitutive promoters could be that the production of certain polysaccharides (e.g., crystalline polymers) could be detrimental to the growth of the orthogonal host and might hence reduce biomass accumulation. Decoupling growth from production formation enables more precise characterization of the polymer formation over time. Although the same regulatory elements (promoter and transcriptional terminator) can be re-used for several genes in a biochemical pathway, repetitive elements increase the risk of rearrangements (due to homologous recombination) and/or gene silencing, at least in yeast and bacteria. For instance, in yeast, this challenge can be addressed via the use of distinct promoters [27], or by using a single promoter to express multiple proteins separated by self-processing viral 2A peptide sequences [28]. 

Transgenes can be expressed using self-replicating plasmids (episomal), which requires active use of one or more selection markers (e.g., an antibiotic), or stably integrated in the genome of the host. The latter approach has the advantage that the transgene will be genetically inherited without active selection. While auxotrophy markers (e.g., leucine) can be used with specific strains (e.g., leucine-deficient), antibiotic selection markers are dominant and can be applied more broadly [29]. The number of selection markers compatible with a host strain proportionally increases the rounds of transformation that can be performed to sequentially introduce new vectors or transgenes.

In addition to the genetic considerations outlined above, an orthogonal host for polysaccharide production should not contain endogenous polymers that resemble the target product to avoid analytical interference and thus simplifying the detection of the product through various methods. Conversely, the composition of native glycans reflects the potential availability of nucleotide sugars, which is an important attribute that has not been extensively characterized in the selected species. To assess the polymer structures present in the extracellular matrix of various potential hosts, we performed a glycosidic linkage analysis (Table 2). Endogenous host polymers were extracted using a procedure used for plant cell wall analysis, commencing with the preparation of an alcohol-insoluble residue (AIR) removing soluble, small molecular weight components as well as lipophilic substances. The polysaccharides in the AIR were then derivatized to their partially methylated acetylated alditols followed by gas-chromatographic separation and mass spectrometric detection [37]. This derivatization technique involves an acidic hydrolyzation step. We chose 2 M trifluoroacetic acid, which is widely used for glycosidic linkage analysis of plant polysaccharides. Under these conditions, glucosaminoglycan-containing polymers (e.g., chitin) are usually not hydrolyzed and are not detected or under-represented, thus simplifying the interpretation of the data. Previously, AIR extraction and glycosidic linkage analysis under these conditions have been shown to be sufficient to successfully quantify the production of plant hemicellulose backbones in a yeast [17,38]. In the next three sections, we review the genetic and glycomic attributes of the orthogonal hosts considered from the kingdoms of Bacteria, Fungi and Animalia.

## 3. Bacteria

Bacteria have the advantages of rapid doubling time and low cultivation costs, but they are prokaryotes lacking an endomembrane system and several PTMs (such as protein glycosylation) present in plants (Table 1). Two bacterial species (*E. coli* and *B. subtilis*) have a larger number of strains and vectors available to order than the other organisms summarized in Table 1. Compared to eukaryotes, bacteria are also simpler to transform and manipulate (e.g., for direct evolution studies), thereby shortening the experimental time required to go from gene to function or product. Despite these advantages, there are very limited successful efforts to study the activities of plant CWGTs expressed in bacterial systems. Previously, *Arabidopsis thaliana* GT34 family proteins were expressed as GST-fusion proteins in *E. coli* and three members from the XXT clade showed xylosyltransferase activity in vitro [39]. A high-throughput expression screen for a total of 46 CWGTs from several plant species found that the correct folding of the recombinant proteins was problematic in *E. coli*, but could be partially improved by co-expression with chaperones [40]. Despite the potential of the screening pipeline, only one of the enzymes tested in this screen (Reversibly Glycosylated Polypeptide 1, RGP1), which natively lacked a transmembrane domain, was produced at the scale and purity needed for molecular studies. In contrast to the in vitro studies, the synthetic biological approach outlined in Figure 1 would not be impacted by the extractability of the heterologous enzymes, if they are correctly folded and functional in the orthogonal host.

### 3.1. Gram-Negative Bacteria

The most commonly used bacteria for protein expression is *E. coli* (Figure 2). This bacterium stains gram-negative indicating that in addition to a peptidoglycan layer, it is often surrounded by diverse layers of surface polysaccharides (e.g., a lipopolysaccharide or a capsular polysaccharide [41]). The composition of these polysaccharides is highly variable and usually isolate-specific. Linkage analysis of the *E. coli* DB3.1 strain analyzed here indicated the presence of a galactose- and ribose-rich extracellular matrix (Table 2). These are likely components of the capsular polysaccharide described for some *E. coli* strains [42,43]. A toxigenic strain of *E. coli* has been shown to produce cellulose, under growth conditions that lead to biofilm formation [44]. It is noteworthy that non-toxigenic *E. coli* cells were recently engineered to secrete large bacterial cellulose fibers into the culture media, after expressing six recombinant proteins from the *Gluconacetobacter hansenii* (also known as *Acetobacter xylinum*) gram-negative bacterium [45]. Despite its low abundance (Table 2), the 4-Glc found in the growth conditions used here might be derived from cellulosic polymers that encapsulate *E. coli* cells. 

*Pseudomonas fluorescens*, another gram-negative bacterium, has been used for agricultural applications and therapeutic proteins in the last two decades [46]. *Pseudomonas fluorescens* is reported to have less strict fermentation processes compared to *E. coli*, and was superior for the yield and solubility of certain recombinant proteins [47]. Antibiotic as well as auxotrophic markers, several inducible promoters, rapid cloning vectors based on Type IIS restriction sites and a variety of host strains (e.g., protease-deficient mutants) have been established for *Pseudomonas* [46]. Despite these genetic advantages, a significant drawback is that *Pseudomonas fluorescens* subsp. *cellulose* can secrete cellulase, xylanase, mannanase enzymes and additional glycosyl hydrolases that enable this bacterium to be cultivated on plant polysaccharides (e.g., crystalline cellulose, xylan or galactomannan) as its main carbon source [48,49]. It is unclear how prevalent these activities would be in other *Pseudomonas* varieties, but a recently established CRISPR interference system could be used to repress the endogenous genes that are detrimental for plant polysaccharides synthesis [50].

The extracellular matrix of *Pseudomonas fluorescens* seems to consist of a branched glucan (mainly 6-linked) and a branched rhamnan consistent with the production of a rhamnose-rich lipopolysaccharide in this bacteria, as described for other *Pseudomonas* species [51]. Therefore, the *Pseudomonas* wall composition would only interfere with plant rhamnogalacturonan production. Other plant polysaccharides would not be masked by native *Pseudomonas* wall components but may be digested by endogenous hydrolases.

### 3.2. Gram-Positive Bacteria

*Bacillus subtilis* is a frequently used gram-positive expression system that secretes numerous enzymes of commercial value such as α-amylases to degrade starch [52]. Thanks to its industrial relevance and its engineering as a host for synthetic biology, *B. subtilis* is regarded as a “super-secreting cell factory” [53]. Due to the lack of an outer membrane system, the wall polymers produced in such cells might be more easily accessible. However, wild-type *B. subtilis* and other *Bacillus* species participate in the microbial degradation of the plant cell wall and have been used to isolate and characterize enzymes that degrade matrix polysaccharides including heteromannan [54], and various domains of pectin (RG I [55], homogalacturonan [56], arabinan [57], galactan [58]). Since *B. subtilis* strains with reduced protease activity have been constructed [52], a similar strategy could work for glycosyl hydrolases. In contrast to *E. coli*, we could not find any published examples of plant GT expression in *B. subtilis*. In terms of wall composition, *Bacillus* species, such as *B. anthracis* [59], produce diverse polysaccharides that are often comprised of a repeating trisaccharide with galactosyl modifications. The cell wall linkage analysis of *B. subtilis* performed here indicates the presence of a galactan, with some mannoproteins, and a ribose containing polymer. There is a high proportion of terminal glucose indicative of many non-reducing glucose ends. Since none of these glycosidic linkages would interfere with the production of a eukaryotic polysaccharide, the secretion of endogenous glycosyl hydrolases capable of degrading plant cell wall components represents a greater concern. The low amount of native cellulolytic activity of *B. subtilis*, previously exploited as a platform for recombinant cellulase expression [60], suggests that this host might be suitable to produce plant cellulose.

### 3.3. Cyanobacteria

Cyanobacteria have the advantage of being photoautotrophic and have thus attracted interest for the production of renewable fuels and other small molecules directly from CO_2_ [61]. We evaluated the suitability of *Synechocystis* PCC 6803 and *Synechococcus elongatus* sp. PCC 7942, the two species with the most advanced metabolic engineering record in the cyanobacterial field [61]. Compared to the other classes of bacteria discussed in this review, the growth of cyanobacteria is enhanced by photosynthetically active light radiation and elevated CO_2_. Cyanobacteria can be cultivated for biomass accumulation in open systems (e.g., ponds) or in closed systems (photobioreactors) [62], similar to microalgae [63]. While *Synechococcus* UTEX 2973 is the record holder among cyanobacteria with a 1.9 h doubling time, its better studied relative *Synechococcus elongatus* PCC 7942 has only a 4.1 h growth rate [64]. There may be room to further improve the growth rates of cyanobacteria, since medium optimization for extended cultivation enhanced the doubling time of *Synechocystis* from the textbook value of 8 h to only 4.3 h [35]. Several promoters for recombinant protein expression, including some adopted from *E. coli* [65], are available for *Synechocystis*, and a library of inducible promoters has now been evaluated [66]. The discovery of a “super-strong” promoter enables cyanobacteria to be considered as alternative hosts for heterologous protein expression [67], an application for which they were historically neglected. Moreover, a CyanoGate modular cloning system based on the MoClo syntax is now publicly available for cyanobacteria [68]. Despite no published attempts to express plant CWGTs in either host, efforts have been made to glycoengineer the thylakoid membranes of cyanobacteria [69], and to increase photosynthetic carbon partitioning towards desired metabolites, notably terpenoids [70]. 

Although cyanobacteria are considered gram-negative bacteria due to the presence of an outer membrane system, their cell wall contains features of gram-positive bacteria such as a thicker peptidoglycan layer. From a polysaccharide perspective, their composition can vary depending on the species. Based on the linkage analysis, *Synechococcus’* wall contains a branched glucan, a mannan, and even galactosyl- and xylosyl residues (Table 2) consistent with another study that evaluated the monosaccharide composition of *Synechococcus* biomass as a feedstock for yeast fermentation [71]. Hence, *Synechococcus* would not be a favorable host for the production of the heteromannan or heteroxylan hemicellulosic polymers. The 4-linked glucose linkage indicative of cellulose is present only in low amounts. This was already leveraged to heterologously express cellulose synthase genes from the *Acetobacter xylinum* bacterium, which resulted in the production of non-crystalline, extracellular cellulose in *S. elongatus* sp. PCC 7942 [72]. Similarly, the cyanobacterium *Synechococcus* sp. PCC 7002 was shown to naturally contain cellulose but secreted very large amounts of extracellular cellulose after overexpression of *A. xylinum* enzymes [73].

In contrast to *Synechococcus* (Table 2), the *Synechocystis’* wall seems to contain a 4-fold higher proportion of 4-linked glucose, likely derived from cellulose. In addition, branched glucosyl-, xylosyl- and a low proportion of mannosyl residues are present (Table 2). These sugar moieties have been shown to be present in the exopolysaccharide of the *Synechocystis* 6803 strain also analyzed here [74]. Hence, this organism could be used for heteromannan production.

## 4. Fungi

### 4.1. General Evaluation of Four Species

As eukaryotes, fungal cells contain an endomembrane system that features similar organelles to those found in more complex organisms such as plants. This is a key advantage over bacterial hosts, since the endomembrane system provides the sites for the elongation and substitution of wall polysaccharides in plant cells. Compared to bacteria, fungi are the simplest organisms to feature endogenous PTMs such as glycosylation which are important for enzyme activity in higher eukaryotes. In addition to providing suitable compartments for heterologously expressed plant CWGTs, fungi also supply some of the required substrates for enzyme activities in the desired places. Like plants, yeast produce activated nucleotide sugars such as UDP-Glucose and GDP-mannose in the cytosol, and have endogenous transporters capable of transporting these substrates to the Golgi lumen [75]. Indeed, the availability of nucleotide sugar transporter mutants in yeast [76] enabled the functional analysis of the Golgi-localized homologs in plants [77]. Depending on their number of transmembrane spans and orientation, polysaccharide synthases could have an active site facing the Golgi lumen (as proposed for glucomannan synthases), or may face the cytosol and translocate the product across the membrane (e.g., xyloglucan synthases) [78]. 

Although *Saccharomyces cerevisiae* is the best characterized yeast and has been at the forefront of synthetic biology since its inception (Figure 2), only plant GTs involved in secondary metabolism [79], but no plant CWGTs, have been expressed in this host. In contrast, *Pichia pastoris* (formally reclassified as *Komagataella phaffii*, but simply called *Pichia* in this review) has emerged as a convenient host for the expression and characterization of plant CWGTs, such as RGXTs [80] and CSLAs [81], whose biochemical activities were first elucidated using insect cells [20,82]. Compared to cell lines isolated from animals, yeast cells are more convenient to cultivate (speed and cost) and simpler to genetically manipulate (Table 1). Interestingly, *Pichia* is also the first and so far the only orthologous host to express plant cellulose synthases that are functional in vitro [83,84]. Furthermore, unbranched glucan [38] and (gluco)mannan polysaccharides [17] have already been synthesized in *Pichia* using the strategy outlined in Figure 1. In addition, the number of molecular biology tools available for “non-conventional” yeast species, such as *Pichia* and *Yarrowia lipolytica*, is rapidly increasing. Several collections of versatile vectors are now available from the AddGene repository for *Pichia* (GoldenPiCS [27] and MoClo Pichia toolkit [85]), and *Yarrowia lipolytica* (YaliBricks [86], GoldenMOCS [87], and EasyCloneYALI [88]) featuring constitutive and inducible promoters, a variety of transcriptional terminators, and selection markers. Compared to *Saccharomyces*, which hyperglycosylates eukaryotic recombinant proteins with up to 200 mannose units, *Pichia* and *Yarrowia* do this to a lesser extent (~20 mannose) [89] and have both been engineered to produce designer N-glycans for therapeutic recombinant proteins [30,90].

In general, *Saccharomyces* [91], *Pichia* [17,38] and *Yarrowia* [92] share similar cell wall constituents: β1,3- and β1,6-linked glucans, mannoproteins, and small amounts of chitin. Since polymers with a high degree of crystallinity (e.g., chitin) are resistant to the acid hydrolysis conditions used in this study, their abundance is likely under-represented in our glycosidic linkage analysis (Table 2). Our *Saccharomyces* data indicates the presence of a branched glucan, but very little 4-linked glucose or mannose linkages interfering with the analysis of cellulose or plant mannan. Compared to *Saccharomyces*, *Pichia* adds fewer mannose residues to N-glycans [89], and its wall contains branched glucans and some branched mannoproteins (Table 2). The wall preparation also contains significant amounts of 4-linked glucose likely derived from storage glycogen. Hence, the production of cellulose and glucan containing polymers such as mixed-linkage glucan and xyloglucan might be difficult to discern in *Pichia*. Interestingly, *Yarrowia* was confirmed to have endogenous enzymes and transporters required for Gal metabolism [93], which *Pichia* lacks. This feature has indeed been confirmed by our glycosidic linkage analysis, where terminal galactosyl-residues were found in the *Yarrowia* wall (Table 2). This might aid in the synthesis of galactose-containing plant polymers, although the terminal-galactose units could partially mask galactomannan production (Table 2). 

In addition to the three yeast species discussed above, we also assessed the suitability of *Ustilago maydis*, a smut fungus that has been used as a system to study plant cell wall degrading enzymes. As noted earlier in the review, non-pathogenic strains of *Ustilago* have already been engineered [94], including the haploid AB31 variety analyzed here. In general, the wall of *Ustilago* is similar to the other yeasts [95]. Based on the glycosidic linkage analysis, there is a high abundance of 4-linked glucose, likely glycogen (Table 2), and only a minor amount of mannoproteins. Interestingly, *Ustilago* contained similar levels of terminal-galactose to *Yarrowia*. However, *Ustilago* was the only host examined that contains 4,6-glucosyl residues, which would interfere with xyloglucan production. There are also ribose-containing polymers, which would not affect plant polysaccharide production.

### 4.2. Direct Comparison of Two Hosts

**Figure 3:**
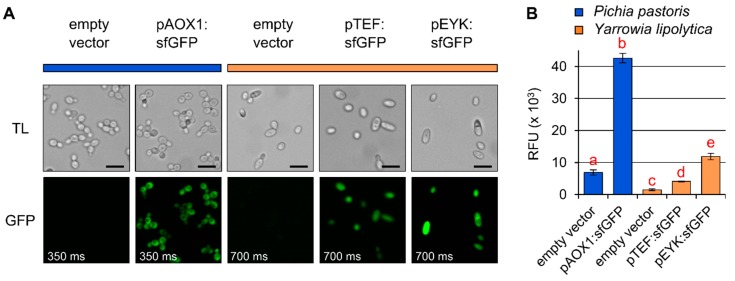
Comparison of fluorescent protein expression in two different yeast species. *Pichia* (blue) and *Yarrowia* (orange) cells after 24 h of growth in methanol- or erythritol-containing media (see Supplemental Materials and Methods), respectively, and expressing no transgene (empty vector) or sfGFP under control of the methanol-inducible *pAOX1*, the constitutive *pTEF*, or the erythritol-inducible promoter *pEYK*. (**A**) Widefield micrographs of transmitted light (TL) and GFP signals in yeast cells after exposure (time indicated in each panel) to a halogen light source. Scale bars = 10 µm. (**B**) Relative fluorescence units (RFU; normalized to optical density at 600 nm) of yeast cells quantified with a plate reader. Different letters indicate significant changes based on one-way ANOVA with post hoc Tukey HSD Test (*P* < 0.05). Data show mean ± SD of three biological replicates.

Although recombinant protein expression has been tested in all the organisms outlined in Table 1 and Figure 2, there are relatively few side-by-side comparisons and pilot experiments are likely required to compare two species for a specific application. As a proof-of-concept, we directly compared the suitability of *Pichia* and *Yarrowia* using the superfolder green fluorescent protein (sfGFP) as a reporter (Figure 3). In *Pichia*, the transgene was expressed under control of the methanol-inducible *ALCOHOL OXIDASE 1* promoter (*pAOX1*), which is readily available in a range of vectors from AddGene or commercial suppliers (e.g., Invitrogen, Thermo Fischer Scientific). For *Yarrowia*, we tested the common *pTEF* promoter, which is constitutive [96], and the *pEYK300A3B (pEYK)* hybrid promoter, which is erythritol-inducible and is reported to result in five-fold higher expression than *pTEF* [97]. After direct cultivation in an appropriate medium containing the inducer, both yeast species showed sfGFP fluorescence when the transgene was expressed compared to empty vector controls (Figure 3A). The relative fluorescence of sfGFP proteins expressed in *Pichia* was visibly stronger than in *Yarrowia*, and hence a two-fold lower exposure time was used to avoid signal saturation in the *Pichia* micrographs. Furthermore, the relative fluorescence of the sfGFP recombinant protein was quantified with a plate reader and normalized to the optical density (OD600) of the cells (Figure 3B). Based on our cultivation conditions and the sfGFP reporter protein, we observed a three-fold increase in fluorescence intensity using *pEYK* instead of *pTEF* (Figure 3B). It is noteworthy that despite the advantage of *Yarrowia* for the synthesis of galactose-containing polymers (Table 2), the relative intensity sfGFP expressed in *Pichia* under the control of the *pAOX1* promoter was several fold higher than even the *Yarrowia pEYK* construct. An additional consideration for many *Yarrowia* strains is that *zeta* elements found in popular vectors enable random chromosomal integration [98], which results in varying levels of protein expression. A 2018 study also showed that *Yarrowia* can also efficiently integrate multi-gene biochemical pathways in a homology-independent manner [99]. Despite the need to screen a larger number of colonies than in *Pichia*, a benefit of this random integration is that clones with different expression levels can be isolated with a single promoter and that subsequent rounds of transformation are unlikely to integrate in the same locus, thus enhancing pathway engineering. 

## 5. Animal Cells

Immortal animal cell lines such as Chinese ovary hamster (CHO) and HEK293 cells have received attention as model systems for synthetic biology particularly for mammalian protein production (Figure 2). These cells are prized to produce therapeutic proteins without the hyperglycosylation issues found in yeast. Animal cells have also found expanded use for the characterization of recombinant plant CWGTs that were challenging to purify in sufficient quantities in other orthogonal hosts. For example, HEK293 cells have been successfully used to express active pectin homogalacturonan galacturonosyltransferases [9,100], xylan synthases [101], and xyloglucan-decorating enzyme XXT1 (for crystal structure determination) [102]. Compared to bacteria and fungi, animal cells are more expensive to cultivate, requiring dedicated growth chambers and complex media. Since glycosylation can have a dramatic impact on antibodies’ properties, glycoengineering of animal cells has already received a lot of attention [103].

The extracellular matrix of animal cells consists mainly of proteins and proteoglycans containing amino sugars, uronic acids and silic acid [104] not found in plant cell walls. Analysis of an extracellular matrix isolated from CHO cells indicates very few glycosidic linkages (Table 2). The dominant component is 4-linked glucose, likely derived from glycogen, and a galactan. For HEK293 cells, we had difficulty in obtaining sufficient material for glycosidic linkage analysis prepared with the method used here. Hence, with the exception of cellulose and other β-glucan containing polymers these hosts are good candidates for the production of plant cell wall polysaccharides, provided that the biomass accumulation is not cost-prohibitive.

## 6. Conclusions

The glycosyl linkage composition of an orthogonal host could be further modified by cultivation on media with different carbon sources. For instance, *Pichia* X-33 shows changes in its native cell wall polymers when grown in the rich YPD medium (Table 2) compared to the buffered minimal media supplemented with glycerol or methanol [17]. Many organisms investigated here contain storage polysaccharides such as starch or glycogen, which under the analysis conditions used result in 4- and 4,6-linked glucose moieties interfering with the presence of cellulose and other glucan-based hemicelluloses such as xyloglucan and mixed linkage glucan. For a clearer interpretation of the results, starch/glycogen can be removed from the AIR preparation with a starch degrading enzyme cocktail prior to derivatization [105]. In the same manner that both animal and glycoengineered yeast cells have merits for antibody production in the pharmaceutical industry [103], we expect that distinct orthogonal hosts could be used to study the biosynthesis of a single class of polysaccharides, particularly to address complementary questions which would be challenging to test in a single species. The genetic tools presented in Table 1 along with the glycosyl linkage analysis of their endogenous extracellular matrix polymers in Table 2 highlight the key advantages and limitations of the various orthologous hosts tested here. As shown in Figure 3, preliminary experimental tests are essential to compare how two or more candidate hosts perform for recombinant protein expression under a defined set of conditions. In addition, the availability of nucleotide sugars and related proteins (such as interconversion enzymes and transporters) could be a limiting factor for glycan biosynthesis in a particular host and requires further investigation. This would be a worthy pursuit since, as shown for bacterial cellulose [72,106,107], the recombinant production of tailored plant polysaccharides could have numerous applications in basic science and biotechnology.

## Figures and Tables

**Table 1:** Summary of the tools available for polysaccharide production in different host organisms. For each species, a relative ranking for different attributes is shown: handling, from least (+) to most convenient (++++); and price, from cheap ($) to most expensive ($$$$). The relative number of strains and vectors available to order were checked at AddGene (www.addgene.org), ATCC (www.atcc.org), DSMZ (www.dsmz.de), Invitrogen, and Sigma-Aldrich. Cultivation costs were based on the complexity of media used, the relative price of components at Sigma-Aldrich, and for the type of incubator used. Doubling times (20 min to 24 h) were estimated based on work by *Pichia* [30], *Yarrowia* [31], *Ustilago* [32], *Pseudomonas* [33], *Bacillus* [34], *Synechocystis* [35], *Synechococcus* [36], and DSMZ (other species). The knockout libraries for different hosts have been generated and are ready to order from AddGene, Dharmacon or AcceGen. The asterisk (*) indicates that the HEK293 mutant collection is not as comprehensive as the rest. “-“ – not described; *E.coli—Escherichia coli* DB3.1; *Pseudomonas fluorescens*; *Bacillus subtilis*; *Synechocystis* PCC 6803: *Synechococcus* *elongatus* sp. PCC 7942; *Saccharomyces cerevisiae*; *Pichia pastoris*; *Yarrowia lipolytica*; *Ustilago maydis;* HEK293 – Human embryonic kidney cells 293; CHO—Chinese hamster ovary cells K1. CWGT—Cell wall-related glycosyltransferase; PTMs—post-translational modifications.

Attribute/Species	*E. coli*	*Pseudomonas*	*Bacillus*	*Synechocystis*	*Synechococcus*	*Saccharomyces*	*Pichia*	*Yarrowia*	*Ustilago*	HEK293	CHO
species classification	Bacteria						Fungi			Animalia	
plant CWGTs expressed	yes	-	-	-	-	-	yes	-	-	yes	-
other plant GTs	yes	-	+	-	yes	yes	yes	yes	-	yes	yes
plant polysaccharide degradation	-	yes	yes	-	-	-	-	-	yes	-	-
eukaryotic PTMs	-	-	-	-	-	yes	yes	yes	yes	yes	yes
available strains	++++	++	++++	+	+	++++	+++	++	+	+++	+++
knockout library	yes	-	yes	-	-	yes	-	-	-	yes*	-
available vectors	++++	+++	++++	++	++	++++	+++	++	+	+++	+++
photosynthetic	-	-	-	yes	yes	-	-	-	-	-	-
cultivation cost	$	$	$	$$$	$$$	$$	$$	$$	$$	$$$$	$$$$
doubling time	++++	++++	++++	++	++	+++	+++	+++	+++	+	+

**Table 2:** Glycosidic linkage analysis of isolated extracellular matrices from various organisms. Organisms were grown as described in Supplemental Materials and Methods and used to determine the composition of the alcohol-insoluble residue (AIR). Shown is the percentage of the total ion chromatogram peak area of the corresponding partially methylated, acetylated alditol representing the linked sugar. Highlighted in blue are glycosyl-moieties that also represent a glycosyl-linkage present in a plant polysaccharide: 3-Glc—mixed-linkage glucan, callose; 4-Glc—starch, cellulose, xyloglucan, glucomannan; 4,6-Glc—starch, cellulose, xyloglucan; 4-Man, 4,6-Man, t-Gal—heteromannan; 4-Xyl—xylan; 2-Rhap, 2,4-Rhap—RG-I. Data show mean ± standard deviation of three biological replicates. “Empty cell”—not detected; a—exact sugar moiety unknown; *E. coli*—*Escherichia coli* DB3.1; *Pseudomonas fluorescens* WCS417r; *Bacillus subtilis*; *Synechocystis* PCC 6803; *Synechococcus elongatus* sp. PCC 7942; *Saccharomyces* *cerevisiae* BY4742; *Pichia pastoris* X-33; *Yarrowia lipolytica* Po1d; *Ustilago maydis* AB31; CHO—Chinese hamster ovary cells K1.

	*E. coli*	*Pseudo-* *monas*	*Bacillus*	*Synecho-* *cystis*	*Synecho-* *coccus*	*Saccharo-* *myces*	*Pichia*	*Yarrowia*	*Ustilago*	CHO
**t-Glc**	4.4 ± 0.4	5.5 ± 0.3	12.9 ± 2.7	11.5 ± 0.2	1.6 ± 0.2	42.2 ± 2.5	8.0 ± 1.0	6.6 ± 1.7	7.4 ± 0.1	4.4 ± 0.7
**3-Glc**				1.9 ± 0.0	51.8 ± 3.1	4.9 ± 0.7	5.3 ± 1.4	1.9 ± 0.6	8.9 ± 0.7	
**6-Glc**		15.2 ± 0.7				7.0 ± 1.2	7.8 ± 1.6	10.0 ± 1.2	10.8 ± 1.0	
**2,3-Glc**				0.7 ± 0.0		2.3 ± 0.1	2.1 ± 1.0	0.4 ± 0.1	0.5 ± 0.1	
**3,6-Glc**		1.6 ± 0.0			4.2 ± 0.5	2.5 ± 0.4	2.3 ± 0.6	0.7 ± 0.4	4.5 ± 0.2	6.3 ± 0.6
**3,4-Glc**				24.1 ± 0.7						
**4-Glc**	2.7 ± 0.1	1.5 ± 0.4	2.9 ± 0.8	12.2 ± 0.2	2.9 ± 1.1	3.9 ± 0.4	14.3 ± 3.0	25.5 ± 3.8	30.4 ± 0.9	14.3 ± 4.1
**4,6-Glc**									7.0 ± 0.4	
**t-Man**	1.1 ± 0.2		2.1 ± 0.9	0.7 ± 0.0		7.6 ± 1.1	9.3 ± 1.1	7.1 ± 0.2	1.9 ± 0.2	
**2-Man**	5.4 ± 0.3		6.5 ± 2.8	1.5 ± 1.1		11.5 ± 0.5	40.4 ± 0.9	19.2 ± 2.7	1.3 ± 0.1	3.1 ± 0.5
**3-Man**				1.0 ± 0.1						
**4-Man**					21.8 ± 1.6	0.5 ± 0.0	0.3 ± 0.1	0.3 ± 0.1	0.2 ± 0.0	
**6-Man**				1.1 ± 0.1		1.1 ± 0.1	0.8 ± 0.0	8.2 ± 0.8	0.6 ± 0.1	
**4,6-Man**					2.5 ± 0.0		0.0 ± 0.0	0.1 ± 0.0	0.1 ± 0.0	
**2,3-Man**				6.2 ± 0.3	2.9 ± 0.3	1.1 ± 0.1	0.4 ± 0.1	0.2 ± 0.0		
**3,6-Man**						0.6 ± 0.1	1.0 ± 0.2	0.4 ± 0.0	0.5 ± 0.0	
**2,6-Man**				4.4 ± 0.0	4.4 ± 0.4		8.1 ± 0.8	12.3 ± 0.4	0.2 ± 0.1	
**t-Gal**	1.4 ± 0.5		1.9 ± 0.4	4.5 ± 0.2	3.5 ± 0.2			5.7 ± 0.3	6.6 ± 0.1	
**2-Gal**								1.6 ± 0.0		
**3-Gal**	27.0 ± 5.6		35.8 ± 21.6							46.6 ± 1.7
**6-Gal**			30.5 ± 12.9						0.2 ± 0.0	9.0 ± 2.7
**2,4-Gal**		5.5 ± 0.8								
**4,6-Gal**									2.2 ± 0.3	
**t-Rib**			5.5 ± 1.8						9.1 ± 0.9	
**2-Ribf**	34.6 ± 4.7	5.0 ± 0.1				1.1 ± 0.2			0.2 ± 0.0	
**3-Ribf**									7.7 ± 0.4	
**t-Xyl**				0.7 ± 0.2						
**4-Xyl**				25.8 ± 0.8						
**3,4-Xyl**				3.6 ± 0.3	4.5 ± 0.5					
**2,3-Hex^a^**	13.6 ± 0.9									
**2,6-Hex^a^**	5.6 ± 0.6		1.9 ± 0.6			8.1 ± 0.7				
**3,4-Hex^a^**						1.3 ± 0.2				
**3,6-Hex^a^**	2.6 ± 1.3									
**4,6-Hex^a^**						1.3 ± 0.1				
**2,3,4-Hex^a^**						1.7 ± 0.1				
**2,3,6-Hex^a^**	1.7 ± 0.4	1.0 ± 0.1				0.5 ± 0.0				
**3,4,6-Hex^a^**						0.9 ± 0.2				
**t-Rha**		0.5 ± 0.1								
**2-Rha**		15.0 ± 1.6								
**3-Rha**		25.6 ± 1.3								
**3,4-Rha**		13.7 ± 0.6								
**2,3-Rha**		3.5 ± 0.3								
**2,4-Rha**		6.6 ± 1.1								

## Supplementary Materials

The following are available online at https://www.mdpi.com/2223-7747/8/11/516/s1, Supplemental Materials and Methods.
