# Supplementary material for: The Suitability of Orthogonal Hosts to Study Plant Cell Wall Biosynthesis
Source: Plants (Basel). 2019 Nov 17;8(11):516. doi: 10.3390/plants8110516 (PMC6918405; doi:10.3390/plants8110516)
Supplement: Supplementary file 1 [file plants-08-00516-s001.pdf]

Supplemental Material and Methods

Growth conditions

E. coli DB3.1 (Invitrogen), *Pseudomonas fluorescens* strain WCS417r [1], and *Bacillus subtilis* (Sigma 1.10649) were grown in 25mL Luria/Miller media (Carl Roth) for 48h at 225rpm. All bacteria were grown at 28°C except for *E. coli* (37°C). *Synechococcus elongatus* sp. PCC 7942 and *Synechocystis* PCC 6803 liquid cultures were cultivated in BG-11 medium at 30°C, 60 µE light intensity, 75% humidity and shaken at 150 rpm. In addition, the growth chamber was supplemented with 0.5 % CO_2. Yeast (*Saccharomyces cerevisiae* BY4742, *Pichia pastoris* X-33 (Invitrogen) and *Yarrowia lipolytica* A10 [2] were grown in 3mL YPD at 30ºC for 48h at 225rpm. *Ustilago maydis* AB31 was grown in 3mL CM liquid medium 0.25% (w/v) for 24h at 225rpm [3]. Chinese hamster ovary K1 (CHO-K1; DSMZ, Braunschweig, Germany) cells were grown in HAM's F12 medium (PAN Biotech, Aidenbach, Germany) supplemented with 10% (v/v) tetracycline-free fetal bovine serum (FBS; PAN Biotech) and 1.4% (v/v) streptomycin (PAN Biotech). After 4 days of incubation at 37°C and 5% CO_2, cells were harvested.

Preparation of extracellular matrix

The extracellular matrix of the organisms was isolated using a standard plant based alcohol-insoluble residue (AIR) preparation [4]. Briefly, liquid cultures were centrifuged at 4000rpm for 10 minutes and mechanically lysed in 70% aqueous ethanol with glass beads using a Retsch Mill MM400. The residue was retrieved by centrifugation and washed twice with chloroform:methanol 1:1 (v/v) and acetone. The resulting AIR preparation were subjected to glycosidic linkage analysis by gas-chromatography/ mass spectrometric analysis of it partially methylated, acetylated alditols [5]. It should be noted that acid hydrolysis of the methylated polymers was achieved with 2 M trifluoro acetic acid.

Cyanobacteria AIR preparations were treated with α-amylase (Sigma) and pullulanase M2 (Megazyme) in order to remove glycogen. Briefly, AIR was incubated with 15U of α-amylase and 20U of Pullulanase in 0.1M citrate buffer pH 5.0 for 16h at 225rpm and 37°C. After inactivation of the enzymes at 80°C for 10 minutes, treated AIR was collected by centrifugation and washed 3 times with water and once with acetone. Dry glycogen-depleted AIR preparations were used for glycosidic linkage analysis as described above.

The CHO cells were pelleted and after removing the supernatant trypsin-EDTA solution (PAN Biotech) was added to the plates and incubated for 5 min at 37°C. The cell suspensions were transferred to 10 mL fresh HAM's F12 medium supplemented with 10% (v/v) tetracycline-free FBS and 1.4% (v/v) streptomycin and centrifuged for 3 min at 300*g* [6,7]. The extracellular matrix was prepared as AIR as described above.

Yeast transformation

Pichia pastoris was transformed with empty vector pPICZ B as control and pPICZ B vector containing sfGFP [8]. *Yarrowia lipolytica* was transformed with empty vector E3 vector (JMP62 URA3 ex_pTEF [9]), or the E3 vector expressing sfGFP under the control of constitutive promoter TEF and modified E3 vector expressing sfGFP under the control of the erythritol-inducible promoter pEYK. The hybrid promoter pEYK300A3B (pEYK) was synthesized commercially and used to replace the pTEF promoter using Bsu15I and BamHI restrictions. For the data in Figure 2, *Pichia* was grown directly in methanol-containing complex medium (as described in the Pichia Easy Select Manual, Invitrogen) while *Yarrowia* was grown in erythritol-containing minimal

medium (YNBE; [10]) for induction. Yeast cultures were inoculated in 600 µL of the appropriate medium in a 24-well plate (VWR, 734-2779), and incubated at 30 °C and 250 rpm for 24 h. Micrographs of the yeast cells were made with a black and white camera (Zeiss AxioCam MR R3) equipped on an Axioplan 2 fluorescence microscope (Zeiss) and the Alexa Fluo 488 filter for GFP fluorescence. Images were processed uniformly in Fiji [11].

Supplemental Bibliography

1. van Loon, L.C.; Bakker, P.A.H.M.; Pieterse, C.M.J. SYSTEMIC RESISTANCE INDUCED BY RHIZOSPHERE BACTERIA. *Annu. Rev. Phytopathol.* **1998**.
2. Barth, G.; Gaillardin, C. Physiology and genetics of the dimorphic fungus Yarrowia lipolytica. *FEMS Microbiol. Rev.* 1997.
3. Brachmann, A.; Weinzierl, G.; Kämper, J.; Kahmann, R. Identification of genes in the bW/bE regulatory cascade in Ustilago maydis. *Mol. Microbiol.* **2001**, *42*, 1047–1063.
4. York, W.S.; Darvill, A.G.; McNeil, M.; Stevenson, T.T.; Albersheim, P. Isolation and characterization of plant cell walls and cell wall components. *Methods Enzymol.* **1986**.
5. Pettolino, F.; Walsh, C.; Fincher, G.B.; Bacic, A. Determining the polysaccharide composition of plant cell walls. *Nat. Protoc.* **2012**, *7*, 1590–607.
6. Baaske, J.; Gonschorek, P.; Engesser, R.; Dominguez-Monedero, A.; Raute, K.; Fischbach, P.; Müller, K.; Cachat, E.; Schamel, W.W.A.; Minguet, S.; et al. Dual-controlled optogenetic system for the rapid down-regulation of protein levels in mammalian cells. *Sci. Rep.* **2018**.
7. Müller, K.; Zurbriggen, M.D.; Weber, W. Control of gene expression using a red- and far-red light-responsive bi-stable toggle switch. *Nat. Protoc.* **2014**.
8. Pédelacq, J.D.; Cabantous, S.; Tran, T.; Terwilliger, T.C.; Waldo, G.S. Engineering and characterization of a superfolder green fluorescent protein. *Nat. Biotechnol.* **2006**, *24*, 79–88.
9. Trassaert, M.; Vandermies, M.; Carly, F.; Denies, O.; Thomas, S.; Fickers, P.; Nicaud, J.M. New inducible promoter for gene expression and synthetic biology in Yarrowia lipolytica. *Microb. Cell Fact.* **2017**, *16*, 1–17.
10. Carly, F.; Vandermies, M.; Damblon, C.; Nicaud, J.M. Identification and characterization of EYK1 , a key gene for erythritol catabolism in Yarrowia lipolytica. **2017**.
11. Schindelin, J.; Arganda-Carreras, I.; Frise, E.; Kaynig, V.; Longair, M.; Pietzsch, T.; Preibisch, S.; Rueden, C.; Saalfeld, S.; Schmid, B.; et al. Fiji: an open-source platform for biological-image analysis. *Nat. Methods* **2012**, *9*, 676–682.
